# A non-conditioned bone marrow transplantation mouse model to study clonal hematopoiesis and myeloid malignancies

**DOI:** 10.1186/s40164-025-00598-8

**Published:** 2025-01-30

**Authors:** Sofia Bentivegna, Marwa Almosailleakh, Lin-Pierre Zhao, Mikkel Bruhn Schuster, Sébastien Benquet, Alexander Balhuizen, Helga Fibiger Munch-Petersen, Lene Dissing Sjö, Mads Hald Andersen, Nicolas Dulphy, Bo Porse, Kirsten Grønbæk

**Affiliations:** 1 Department of Hematology, Rigshospitalet, Copenhagen University Hospital, Copenhagen, Denmark; 2 Biotech Research and Innovation Center (BRIC), University of Copenhagen, Copenhagen, Denmark; 3 Hôpital Saint-Louis, Assistance Publique des Hôpitaux de Paris (APHP), Paris, France; 4 INSERM UMR 1160, Institut de Recherche Saint-Louis, Université Paris Cité, Paris, France; 5 The Finsen Laboratory, Rigshospitalet, Copenhagen University Hospital, Copenhagen, Denmark; 6 Department of Pathology, Rigshospitalet, Copenhagen University Hospital, Copenhagen, Denmark; 7 Department of Clinical Genetics, Rigshospitalet, Copenhagen University Hospital, Copenhagen, Denmark; 8 National Center for Cancer Immune Therapy (CCIT-DK), Department of Oncology, Copenhagen University Hospital, Herlev, Denmark; 9 Department of Immunology and Microbiology, University of Copenhagen, Copenhagen, Denmark; 10 Laboratoire d’Immunologie et d‘Histocompatibilité, Assistance Publique des Hôpitaux de Paris (APHP), Hôpital Saint‐Louis, Paris, France; 11 Institut Carnot OPALE, Institut de Recherche Saint‐Louis, Hôpital Saint‐Louis, Paris, France; 12 Department of Clinical Medicine, Faculty of Health and Medical Sciences, University of Copenhagen, Copenhagen, Denmark

## Abstract

Clonal hematopoiesis of indeterminate potential (CHIP) is a condition where blood or bone marrow cells carry mutations associated with hematological malignancies. Individuals with CHIP have an increased risk of developing hematological malignancies, atherosclerotic cardiovascular disease, and all-cause mortality. Bone marrow transplantation (BMT) of cells carrying CHIP mutations into irradiated mice are useful procedures to investigate the dynamics of clonal expansion and potential therapeutic strategies, but myeloablative conditioning can induce confounding effects. We established a non-conditioned BMT model using C57BL/6J-*Kit*^*W-41J*^/J (W^41^) recipient mice to overcome the unwanted effects of irradiation. Conditional *Tet2* deletion using tamoxifen was used to obtain *Tet2*^*−/−*^ cells from donor mice. Total BM *Tet2*^*−/−*^ cells were transplanted into W^41^ recipients, and longitudinal and terminal analyses at 10 months post-BMT were performed. We showed that W^41^ mice can be used for BMT procedures without myeloablative pre-conditioning. The transplantation of *Tet2*^*−/−*^ BM cells led to a progressive expansion of the donor cells in W^41^ recipients. By modulating the numbers of *Tet2*^*−/−*^ cells transplanted, recipient mice developed features of clonal hematopoiesis or myeloid malignancies. In conclusion, our model is an alternative to conventional irradiation-based transplantation models to study mechanisms underlying malignant hematopoiesis without confounding effects derived from pre-conditioning regimen.

**Supplementary Information:**

The online version contains supplementary material available at 10.1186/s40164-025-00598-8.

**To the editor,**

Clonal hematopoiesis of indeterminate potential (CHIP) refers to a condition characterized by the presence of mutations associated with hematological malignancies, in genes such as *TET2, DNMT3A* and *ASXL1*, in the blood or bone marrow (BM) cells, with a variant allele frequency of 2% or higher, in the absence of a diagnosed hematological disorder [1]. Individuals with CHIP have an increased risk of developing hematological malignancies, and a number of age related disorders including atherosclerotic cardiovascular disease and all-cause mortality [2–4]. Mouse models are invaluable for understanding the molecular and cellular mechanisms of CHIP and hematological disorders, facilitating a deeper understanding of its initiation, progression, and potential therapeutic interventions. Some mouse models rely on BM transplantation (BMT) of donor cells harboring CHIP-associated mutations into irradiated recipient mice [5]. While BMT procedures in irradiated mice have traditionally been employed in the field, this approach has limitations. Irradiation is useful to ablate the recipient’s BM cells, but unwanted effects such as inflammation, alteration of the BM niche, accumulation of BM adipose tissue and damage of several organs accompany this process, which can introduce confounding effects [6–10].

To overcome these limitations, we have established a non-conditioned BMT model using C57BL/6J-*Kit*^*W-41J*^/J mice [11] (referred to as W^41^). These animals harbors a spontaneous point mutation in the *c-Kit* locus that leads to a partial loss of Kit function. This mutation provides a competitive advantage for transplanted *c-Kit* wild type BM donor cells, allowing for minimal or no irradiation [12, 13]. In this study we used non-conditioned W^41^ recipients to transplant *Tet2*-deficient BM cells and we showed that W^41^-*Tet2*^−/−^ chimeras could recapitulate clonal hematopoiesis (CH) and myeloid malignancies.

To create a model that emulates CH, we transplanted 5 million total BM cells, of which 5% were *Tet2* deficient, into W^41^ recipients. We transplanted *Tet2*^+/+^ or *Tet2*^−/−^ (CD45.2) total BM cells together with *Tet2*^+/+^ (CD45.1) total BM cells into unconditioned W^41^ recipients (CD45.1) (Fig. 1A). Longitudinal analysis revealed gradual expansion of *Tet2*^−/−^, but not *Tet2*^+/+^, CD45.2^+^ cells (Fig. 1B). W^41^ mice that received *Tet2*^−/−^ CD45.2^+^ donor cells (referred to as CH mice) showed no differences in total numbers of WBC, RBC or platelets compared to mice that received *Tet2*^+/+^ CD45.2^+^ cells (Fig. 1C). However, at the end of the study, CH mice exhibited significantly less proportion of lymphocytes and higher percentage of neutrophils.

**Fig. 1:**
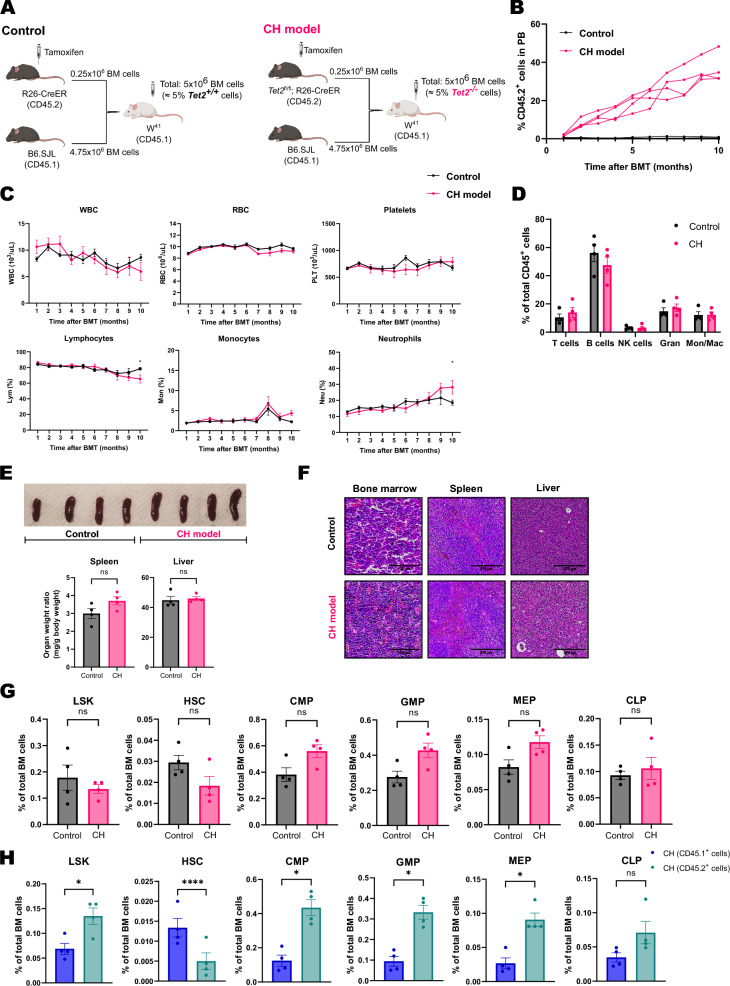
**A** Experimental design. **B** Quantification of the percentage of CD45.2^+^ *Tet2*^+/+^ and *Tet2*^−/−^ cells in PB over 10 months after BMT (n = 4 in each group). **C** Complete blood counts of W^41^ recipient mice transplanted with CD45.2^+^ *Tet2*^+/+^ (n = 4) or *Tet2*^−/−^ cells (n = 4): white blood cells (WBC), red blood cells (RBC). Statistical analysis was performed with two-way ANOVA with Šidák correction for multiple comparisons. Data is presented as mean with SEM. **D** Analysis of PB from control and CH W^41^ mice 10 months after BMT using flow cytometry (n = 4 in each group). Granulocytes (Gran), monocytes/macrophages (Mon/Mac). Analysis was performed using multiple two-tailed t test. **E** Pictures of the spleen from the transplanted W^41^ mice and weight of spleen and liver relative to body weight (Fig. S2A) at the time of analysis. Statistical analysis was done with two-tailed Mann–Whitney U test. **F** Hematoxylin–Eosin (HE) staining of BM, spleen and liver sections. **G** Analysis of hematopoietic stem and progenitor cells (HSPC) in BM of recipient mice using flow cytometry. Statistical analysis was done with two-tailed Mann–Whitney U test. **H** Analysis of CD45.1^+^ (*Tet2*^+*/*+^) and CD45.2^+^ (*Tet2*^*−/−*^) HSPC in BM samples from CH mice. Lin^−^ Sca1^+^ c-Kit^+^ cells (LSK), hematopoietic stem cells (HSC), common myeloid progenitors (CMP), granulocyte/macrophage progenitors (GMP), megakaryocyte/erythrocyte progenitor (MEP), common lymphoid progenitor (CLP). Statistical analysis was done with two-tailed Mann–Whitney U test. The following significance levels were used: ns p > 0.05, *p ≤ 0.05, **p ≤ 0.01, ***p ≤ 0.001, ****p ≤ 0.0001. Bar plots show individual values with mean and SEM

Ten months post-BMT, W^41^ mice were sacrificed, and blood cell populations were analyzed using flow cytometry. Compared to controls, CH mice showed no differences in the proportion of the major cell population analyzed (Fig. 1D and Fig S1). The engraftment of *Tet2*^−/−^ cells did not alter the size of the liver and the spleen relative to body weight (Fig. 1E and Fig. S2A). Histopathological examination of spleen, liver and BM showed no differences between CH and control W^41^ mice (Fig. 1F). Extensive flow cytometry analysis of the BM from transplanted W^41^ mice did not show significant differences in the proportion of different stem and progenitor cells between the two groups (Fig. 1G and Fig. S3). However, there was a significantly higher fraction of CD45.2^+^ LSK and myeloid progenitor cells compared to the competitor CD45.1^+^ cells in CH W^41^ mice (Fig. 1H), and the proportion of total CD45.2^+^ cells in BM was significantly higher than in PB (Fig. S4A). Furthermore, we observed no significant differences between controls or CH mice in the number of colonies obtained after plating total BM cells in methylcellulose for CFU assay (Fig. S5A). Analysis of mature cell populations in BM also showed no differences between the two groups (Fig. S4B), although the percentages of CD45.2^+^ T and NK cells were significantly lower, and the percentage of CD45.2^+^ granulocytes was significantly higher than the CD45.1^+^ counterparts in CH mice (Fig. S4C).

Inactivation of *Tet2* in mice contributes to the development of myeloid malignancies that resemble chronic myelomonocytic leukemia in humans [14, 15]. Therefore, to generate a myeloid malignancy (MM) model, we transplanted 1 × 10^6^ *Tet2*^+/+^ or *Tet2*^−/−^ (CD45.2) total BM cells, without competitor cells, into unconditioned W^41^ recipients (CD45.1) (Fig. 2A), as we found that the presence of competitor CD45.1^+^ cells decreases the number of engrafted *Tet2*^−/−^ cells (Fig. S6). The engraftment and expansion of *Tet2*^−/−^ cells occurred in all the W^41^ mice transplanted (referred to as MM mice) (Fig. 2B). Conversely, we only observed the engraftment of *Tet2*^+/+^ CD45.2^+^ cells in one W^41^ recipient. The expansion of *Tet2*^−/−^ cells induced significant changes in blood parameters, including a reduction in the percentage of lymphocytes and increased proportion of neutrophils and monocytes (Fig. 2C).

**Fig. 2:**
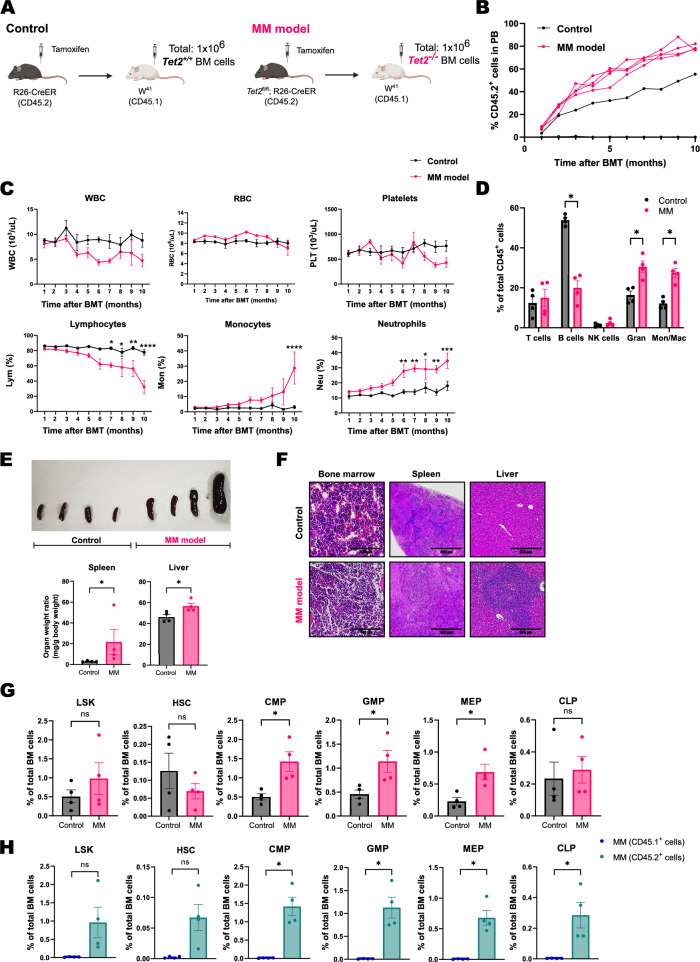
**A** Experimental design. **B** Quantification of the percentage of CD45.2^+^ *Tet2*^+/+^ and *Tet2*^−/−^ cells in PB over 10 months after BMT (n = 4 in each group). The engraftment of *Tet2*^+*/*+^ CD45.2^+^ cells occurred only in one W^41^ recipient (black line). **C** Complete blood counts of W^41^ recipient mice transplanted with CD45.2^+^ *Tet2*^+*/*+^ (n = 4) or *Tet2*^*−/−*^ cells (n = 4): white blood cells (WBC), red blood cells (RBC). Statistical analysis was performed with two-way ANOVA with Šidák correction for multiple comparisons. Data is presented as mean with SEM. **D** Analysis of PB from control and MM W^41^ mice 10 months after BMT using flow cytometry (n = 4 in each group). Granulocytes (Gran), monocytes/macrophages (Mon/Mac). Analysis was performed using multiple two-tailed t test. **E** Pictures of the spleen from the transplanted W^41^ mice and weight of spleen and liver relative to body weight (Fig. S2B) at the time of analysis. Statistical analysis was done with two-tailed Mann–Whitney U test. **F** Hematoxylin–Eosin (HE) staining of BM, spleen and liver sections. **G** Analysis of hematopoietic stem and progenitor cells (HSPC) in BM of recipient mice using flow cytometry. Statistical analysis was done with two-tailed Mann–Whitney U test. **H** Analysis of CD45.1^+^ (*Tet2*^+*/*+^) and CD45.2^+^ (*Tet2*^*−/−*^) HSPC in BM samples from MM mice. Lin^−^ Sca1^+^ c-Kit^+^ cells (LSK), hematopoietic stem cells (HSC), common myeloid progenitors (CMP), granulocyte/macrophage progenitors (GMP), megakaryocyte/erythrocyte progenitor (MEP), common lymphoid progenitor (CLP). Statistical analysis was done with two-tailed Mann–Whitney U test. The following significance levels were used: ns p > 0.05, *p ≤ 0.05, **p ≤ 0.01, ***p ≤ 0.001, ****p ≤ 0.0001. Bar plots show individual values with mean and SEM

Terminal flow cytometry analysis of PB ten months post-BMT revealed a significant reduction of B cells in W^41^ mice engrafted with *Tet2*^−/−^ cells compared to controls (Fig. 2D and Fig. S1). Additionally, MM mice also had a significantly higher proportion of granulocytes and monocytes/macrophages. Compared to the control group, MM mice presented hepatosplenomegaly (Fig. 2E and Fig. S2B). Histopathological examination indicated that the engraftment of *Tet2*^−/−^ cells induced hyperplasia of red and white pulp in the spleen as well as nodular and well-margined infiltrates of lymphocytes in liver sections (Fig. 2F). Multi-parametric flow cytometry analysis of BM from MM mice revealed a significantly higher proportion of myeloid progenitors compared to the control group (Fig. 2G and Fig. S3). Moreover, total BM cells from MM mice plated in methylcellulose formed more colonies, although not at a significant level, than the control counterparts (Fig. S5B). BM samples from MM mice exhibited a significant reduction in B cells compared to the control group (Fig. S7B). It should be noted that the proportion of *Tet2*^*−/−*^ in the BM of transplanted MM mice was nearly 100% at the time of the analysis (Fig. S7A), which explains the significantly higher contribution of CD45.2^+^ cells in several cell lineages (Fig. 2H and Fig. S7C).

In conclusion, our study showed that W^41^ mice can be useful for BMT procedures without pre-conditioning. We showed that when *Tet2*^*−/−*^ BM cells were transplanted into non-irradiated W^41^ recipients, there was a stable engraftment and continuous expansion of Tet2-deficient cells that led to the development of phenotypes that resembled CH or human myeloid malignancies, depending on the number of cells transplanted. Our study showed that BMT procedures in W^41^ mice offer a valuable tool for studying CHIP and hematological malignancies without confounding effects from irradiation.

The use of non-conditioned mouse models have been documented previously to study Tet2-driven CH and its effects on cardiac dysfunction and insulin resistance, which highlights the value of non-irradiated models [16, 17]. In these models, a total of 15 × 10^6^ total BM cells are injected into non-conditioned recipients over three consecutive days. In our work, we showed that by using W^41^ mice as recipients for BMT procedures it is possible to reduce the number of cells injected and the number of injections to achieve engraftment of *Tet2*^*−/−*^ cells.

However, we acknowledge that our study has limitations. TET2 is one of the most frequently mutated genes in patients with CHIP; hence we used murine *Tet2*^*−/−*^ donor cells in our model. Although we have shown that *Tet2*^*−/−*^ cells engraft and expand in W^41^ mice, further studies are necessary to assess whether BM cells carrying different CHIP mutations can be transplanted into these recipients. Furthermore, despite the anticipated competitive advantage of WT HSC over the mutated W^41^ HSC, in our model, we observed that WT cells do not engraft as efficiently as Tet2-deficient cells. Nevertheless, we achieved BM engraftment of WT cells in one animal, suggesting that augmenting the quantity of cells transplanted could facilitate the engraftment of WT cells, and perhaps the engraftment of BM cells carrying mutations in other CHIP-associated genes that do not confer a competitive advantage over WT cells.

## Supplementary Information

Supplementary Material 1.

Supplementary Material 2.

## Data Availability

The data supporting the findings of this study are available within the article and its supplementary information files. Raw files analyzed during the current study are available from the corresponding author upon reasonable request.
