# Supplementary material for: A non-conditioned bone marrow transplantation mouse model to study clonal hematopoiesis and myeloid malignancies
Source: Exp Hematol Oncol. 2025 Jan 30;14:10. doi: 10.1186/s40164-025-00598-8 (PMC11781034; doi:10.1186/s40164-025-00598-8)

Table S1: Antibodies and dyes for PB analysis (BD LSR Fortessa X-20)

**Antibody/Reagent**	**Source**	**Catalog number**
BUV395 Mouse Anti-Mouse CD45.1 (Clone A20)	BD Biosciences	565212
BV605 Hamster Anti-Mouse CD3e (Clone 145-2C11)	BD Biosciences	563004
BV711 Rat Anti-Mouse CD8a (Clone 53-6.7)	BD Biosciences	563046
BV786 Rat Anti-Mouse CD4 (Clone GK1.5)	BD Biosciences	563331
FITC Mouse Anti-Mouse NK-1.1 (PK136)	BD Biosciences	553164
BB700 Rat Anti-Mouse CD19 (Clone 1D3)	BD Biosciences	566411
PE Rat Anti-CD11b (Clone M1/70)	BD Biosciences	557397
PE-Cy™7 Rat Anti-Mouse Ly-6G (Clone 1A8)	BD Biosciences	560601
Alexa Fluor 700 Mouse Anti-Mouse CD45.2 (Clone 104)	BD Biosciences	560693
Fixable Viability Stain 780	BD Biosciences	565388

Table S2: Antibodies and dyes for BM analysis (Cytek AURORA)

**Antibody/Reagent**	**Source**	**Catalog number**
BUV395 Mouse Anti-Mouse CD45.1 (Clone A20)	BD Biosciences	565212
BUV615 Mouse Anti-Mouse CD45.2 (Clone 104)	BD Biosciences	751642
PerCP/Cy5.5 anti-mouse Ly-6A/E (Sca-1) (Clone E13-161.7)	SONY	1212620
PE anti-mouse CD117 (c-Kit) (Clone ACK2)	Thermo Fisher Scientific	12-1172-82
BV650 anti-mouse CD150 (SLAM) (Clone TC15-12F12.2)	SONY	1179660
BUV661 Hamster Anti-Mouse CD48 (Clone HM48-1)	BD Biosciences	741501
BV786 Rat Anti-Mouse CD34 (Clone RAM34)	BD Biosciences	742971
BV421 anti-mouse CD135 (Clone A2F10)	BioLegend	135313
Alexa Fluor 700 CD16/CD32 Monoclonal Antibody (Clone 93)	Thermo Fisher Scientific	56-0161-82
BUV737 Rat Anti-Mouse CD127 (Clone SB/199)	BD Biosciences	612841
APC anti-mouse CD3 (Clone 17A2)	BioLegend	100236
APC-H7 Rat anti-Mouse CD19 (Clone 1D3)	BD Biosciences	560143
Pacific Blue Rat Anti-Mouse CD4 (Clone RM4.5)	BD Biosciences	558107
BV711 Rat Anti-Mouse CD8a (Clone 53-6.7)	BD Biosciences	563046
BV605 Mouse Anti-Mouse NK-1.1 (Clone PK136)	BD Biosciences	563220
PE/Cyanine5 anti-mouse/human CD11b (Clone M1/70)	BioLegend	101210
BUV805 Rat Anti-Mouse Ly-6C (Clone HK1.4.rMAb)	BD Biosciences	755202
PE-Cy™7 Rat Anti-Mouse Ly-6G (Clone 1A8)	BD Biosciences	560601
Zombie UV Fixable Viability Dye	BioLegend	423108

Supplementary figure 1

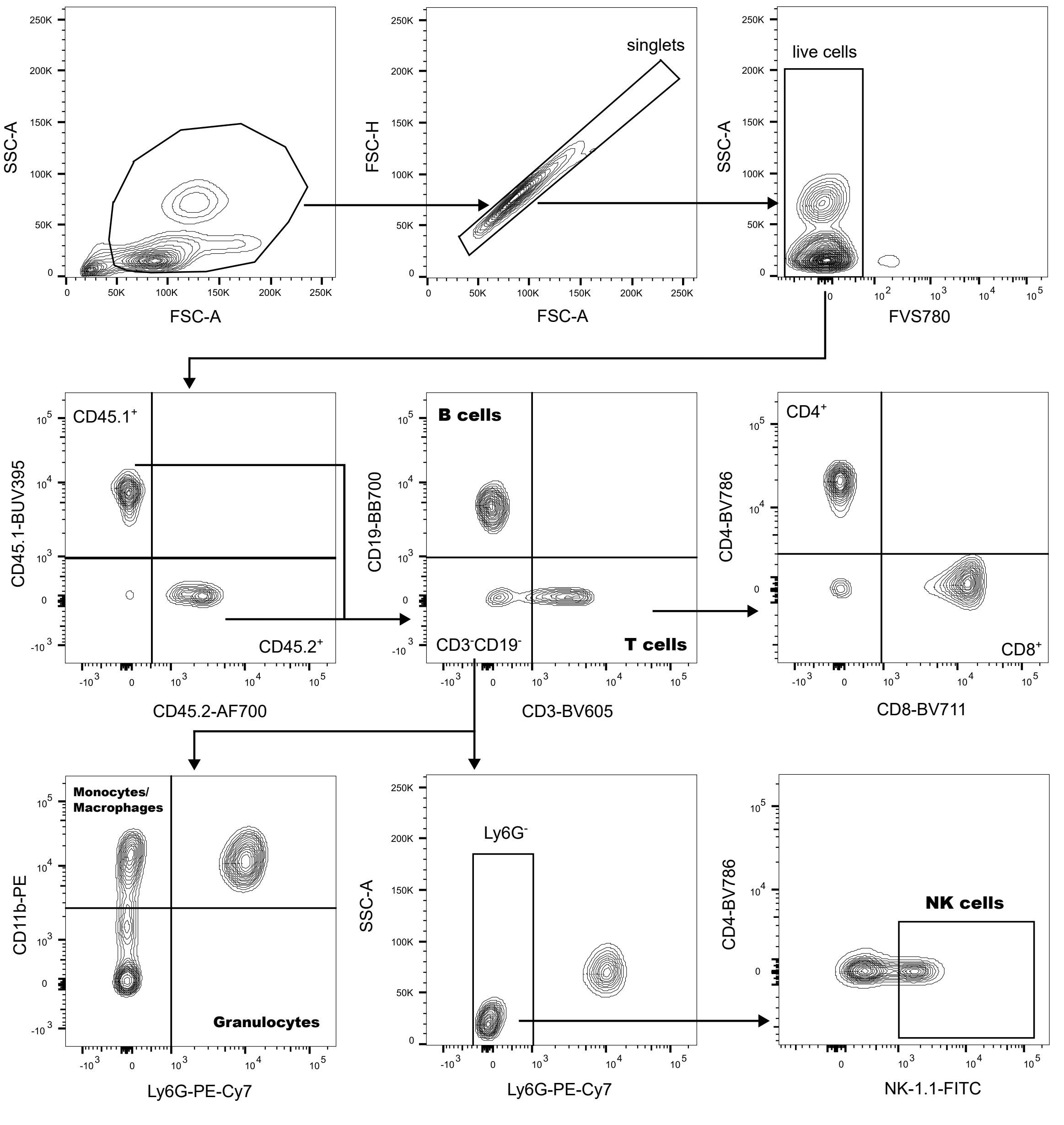

Supplementary figure 2

A

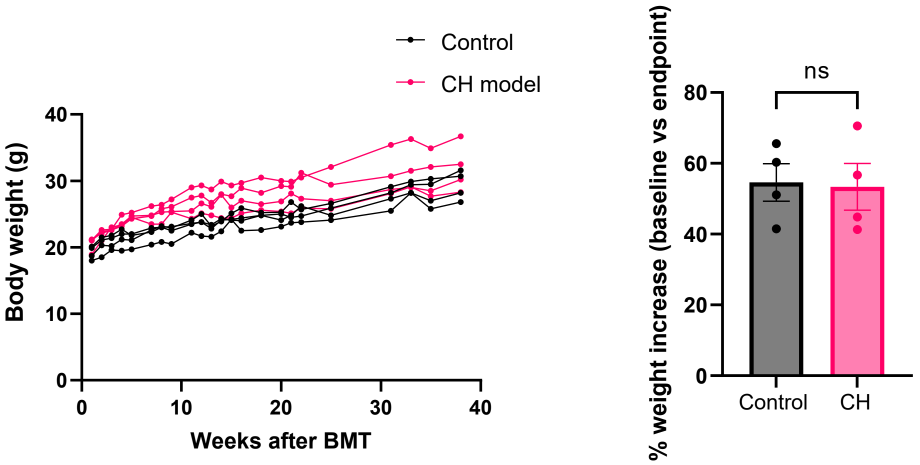

B

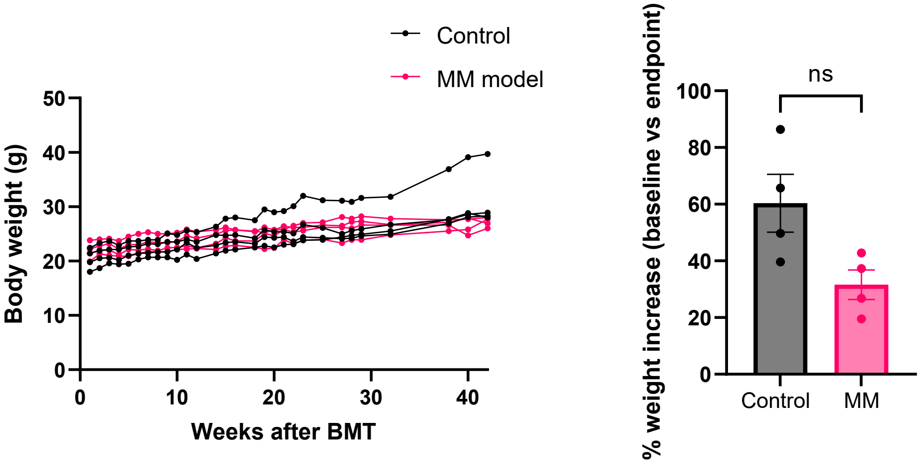

Supplementary figure 3

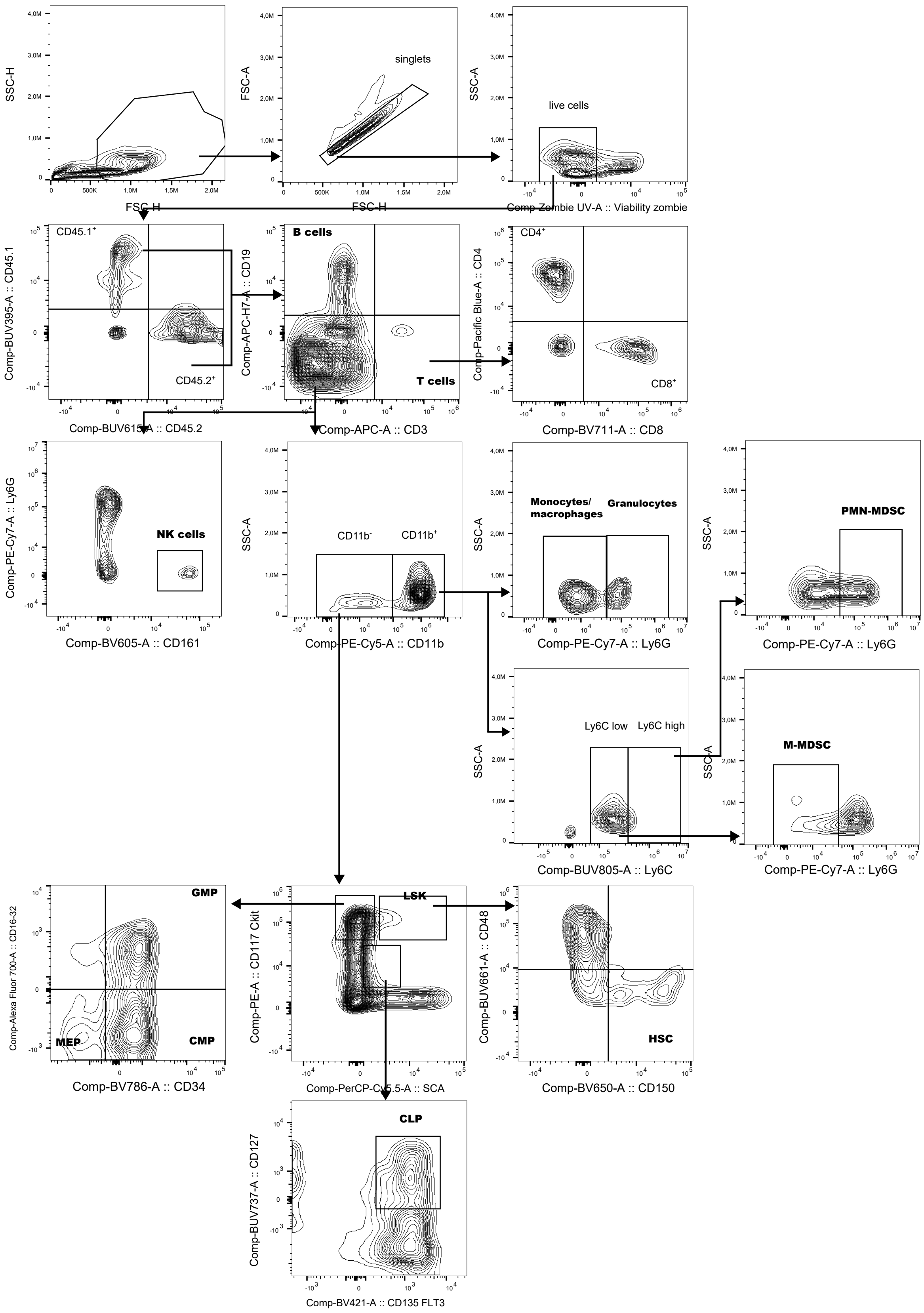

Supplementary figure 4

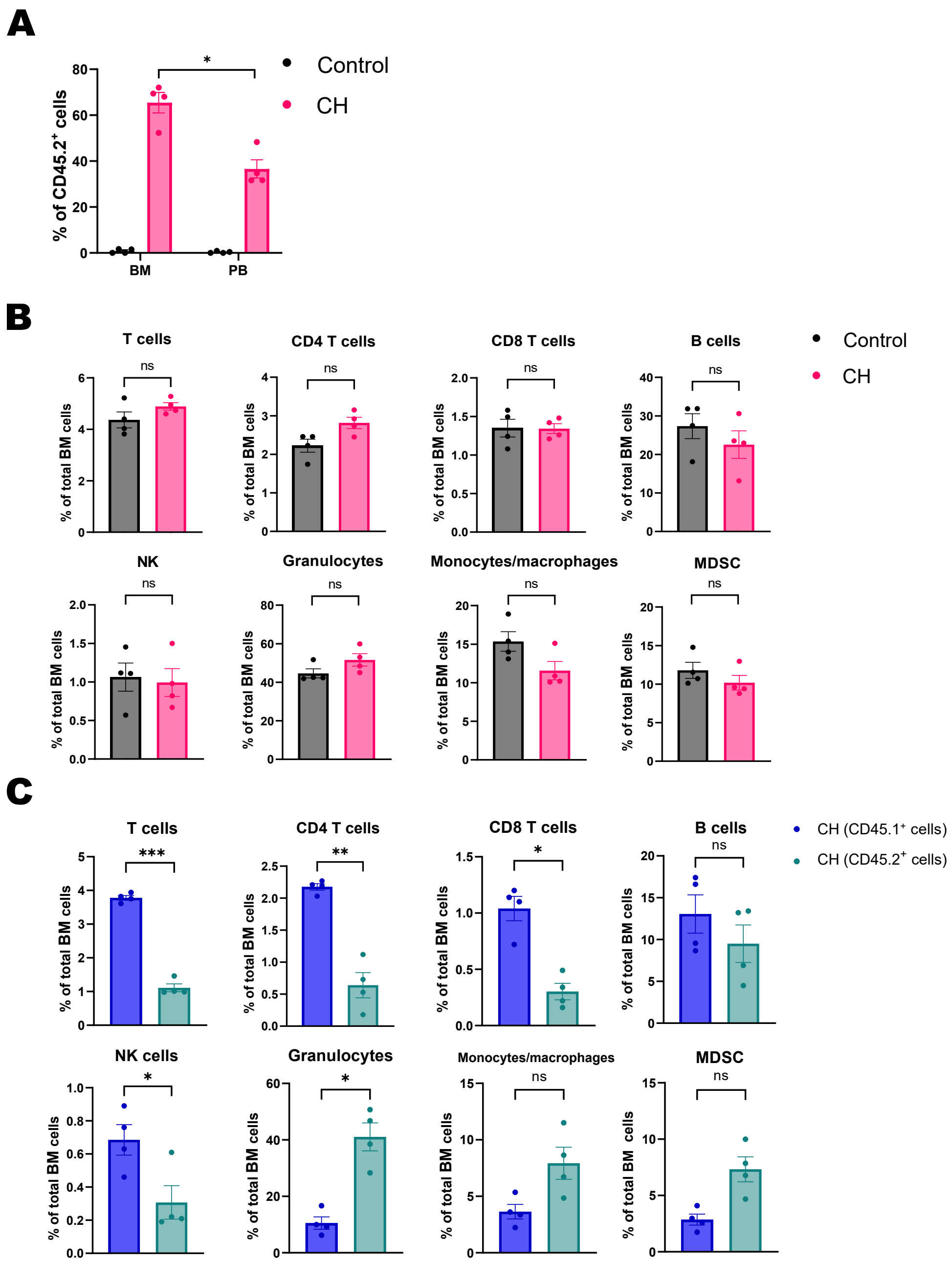

Supplementary figure 5

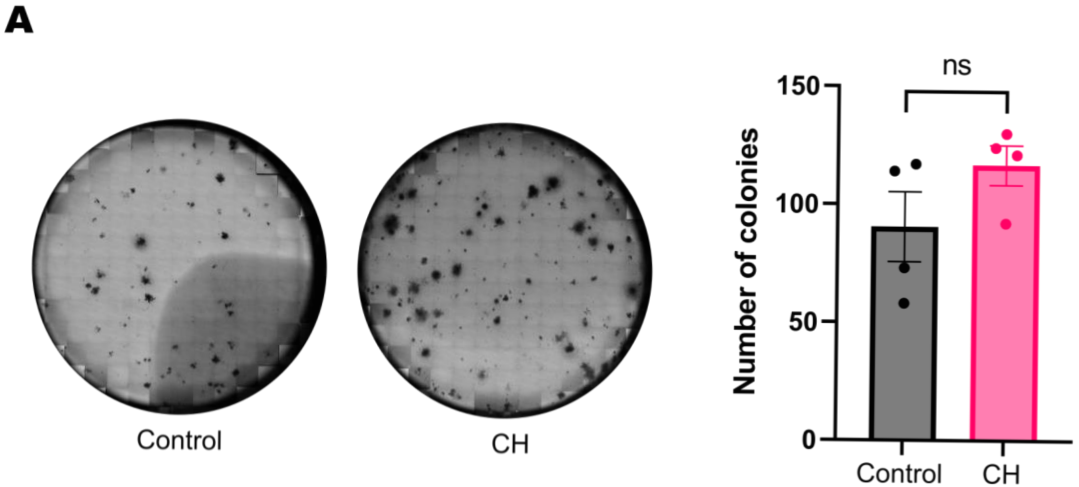

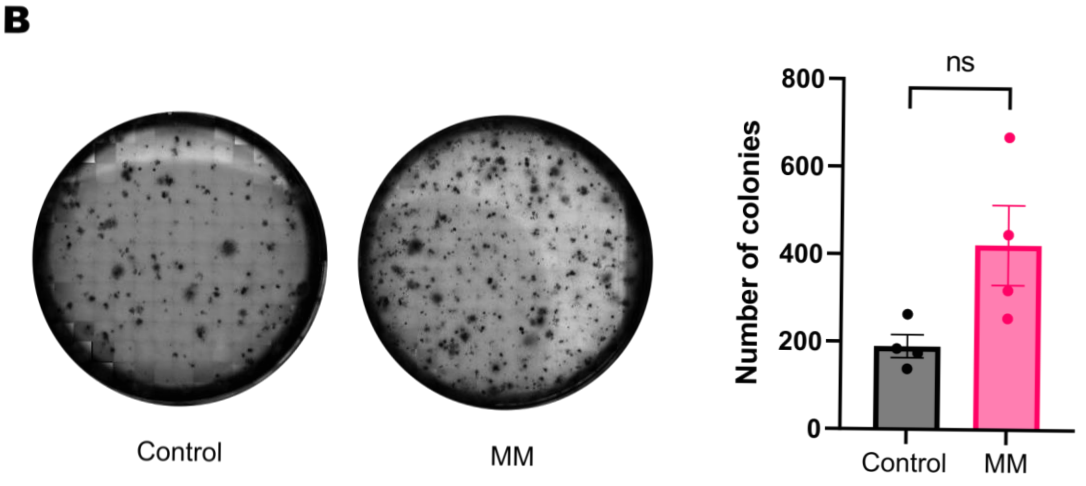

Supplementary figure 6

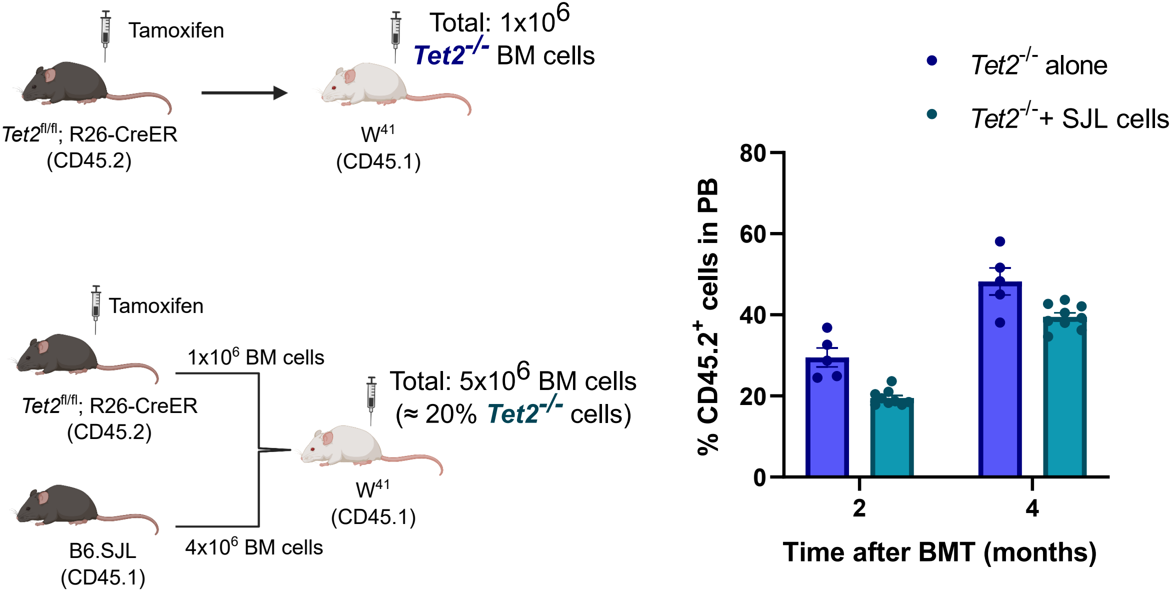

Supplementary figure 7

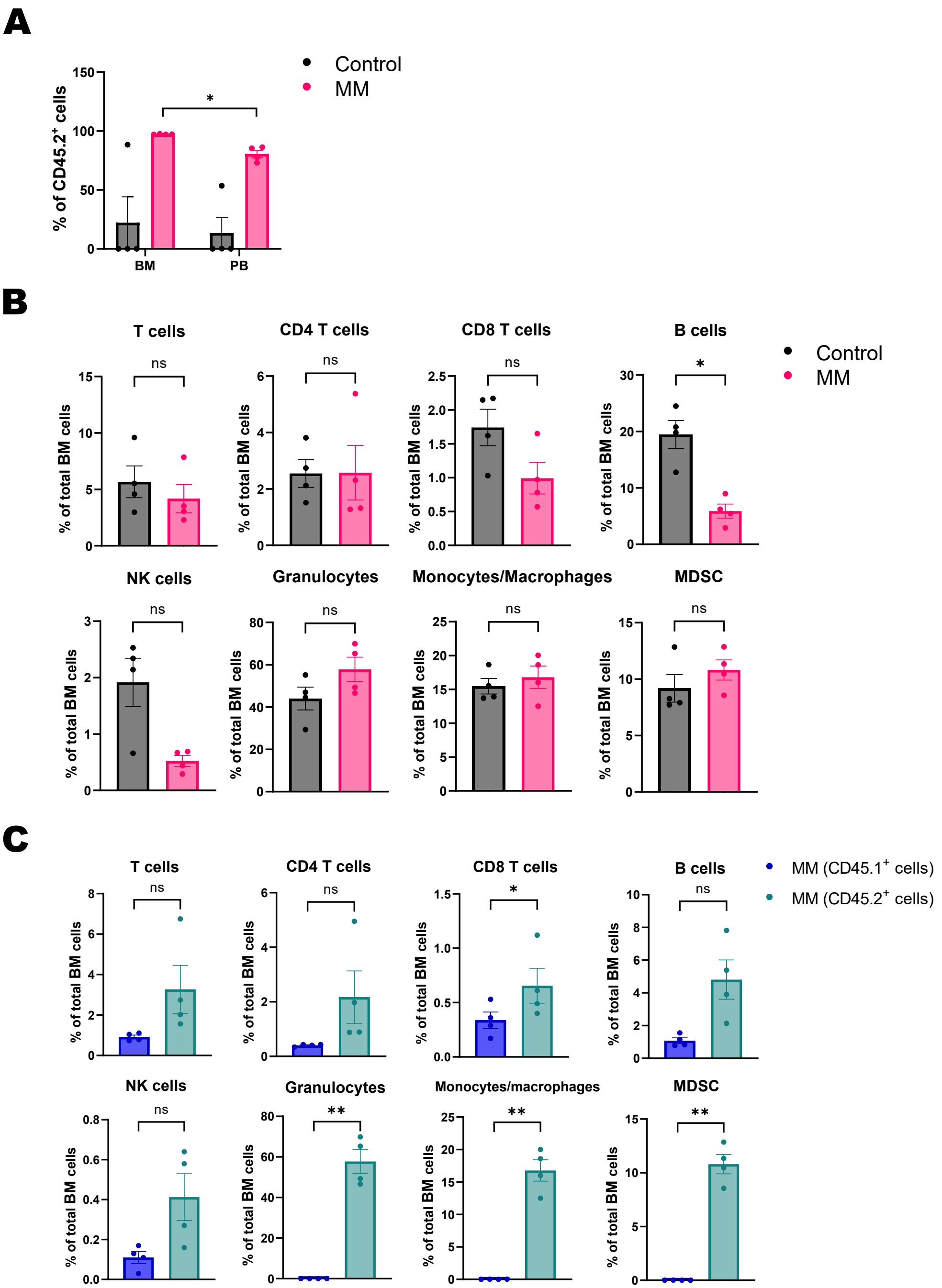

Supplement: Supplementary file 1 — Supplementary Material 1. [file 40164_2025_598_MOESM1_ESM.pdf]
