# Supplementary material for: A non-conditioned bone marrow transplantation mouse model to study clonal hematopoiesis and myeloid malignancies
Source: Exp Hematol Oncol. 2025 Jan 30;14:10. doi: 10.1186/s40164-025-00598-8 (PMC11781034; doi:10.1186/s40164-025-00598-8)
Supplement: Supplementary file 2 — Supplementary Material 2. [file 40164_2025_598_MOESM2_ESM.docx]

**MATERIALS AND METHODS**

Mice

The conditional *Tet2*^fl/fl^ mouse line used was described previously^1^ and was intercrossed with R26-CreER mice^2^ obtained from the Jackson Laboratory. To inactivate *Tet2*, 8-12 week old female *Tet2*^fl/fl^;R26-CreER mice were injected intraperitoneally once every day for 5 consecutive days with 100uL of tamoxifen (Sigma) dissolved in corn oil (Sigma) (20mg/mL). R26-CreER mice received the same treatment and were used as a source of *Tet2*^+/+^ CD45.2^+^ cells. Animals were euthanized 7 days after the final injection. C57BL/6J-*Kit^W-41J^*/J mice were obtained from the Jackson Laboratory and were intercrossed with B6.SJL mice to obtain W^41^ animals harboring the CD45.1 allele. All mouse experiments were conducted according to protocols approved by the Danish Animal Ethical Committee and animals were housed according to institutional guidelines.

BMT

8-12 week old female W^41^ (CD45.1) mice were transplanted through tail vein injection with a suspension of *Tet2*^-/-^ or *Tet2*^+/+^ (CD45.2) total BM cells, with or without BM competitor cells from B6.SJL-Ptprca/BoyAiTac mice (CD45.1). BM cells were obtained from hind limb bones of donor mice after euthanasia. In the CH model, equal numbers of *Tet2*^+/+^ (CD45.2) and *Tet2^-/-^* (CD45.2) cells, with CD45.1 competitor cells, were transplanted into W^41^ mice. Initially, this approach was based on the hypothesis that transplanted cells, irrespective of genotype, would exhibit a proliferative advantage over W^41^-mutated cells and rapidly replace the recipient BM cells. Contrary to this assumption, our experiments revealed that WT cells, when injected in small numbers, could not outcompete the W^41^-mutated cells. As a result, for our MM model, *Tet2*^-/-^ or *Tet2*^+/+^ (CD45.2) cells without additional CD45.1 competitor cells were transplanted into W^41^ recipients.

Blood analysis

For complete blood counts, blood samples were collected in EDTA-coated tubes and analyzed on an element HT5 veterinary hematology analyzer (Heska).

Flow cytometry

For peripheral blood analysis, blood was collected from the facial vein in EDTA-coated tubes. RBC lysis was performed using ACK Lysing Buffer (Gibco). For BM analysis, hind limb bones were harvested, crushed using mortar and pestle and resuspended in phosphate buffed saline (PBS) with 2% fetal bovine serum (FBS). Cells were kept frozen and they were thawed for the analysis.

Fc-receptor blocking was performed prior to antibody staining using purified Mouse BD Fc Block™ (BD Biosciences). Cell were then incubated in PBS with 2% FBS containing Brilliant Stain Buffer Plus (BD Biosciences) with antibody cocktails for 20 minutes at room temperature, then washed with PBS and fixed using Fixation Medium (Medium A) (Invitrogen). Details about the antibodies used can be found in the supplementary material (Tables S1 and S2). Samples were analyzed on BD LSR Fortessa X-20 or in a Cytek AURORA. Data was analyzed using FlowJo 10.8 (Becton Dickinson).

Frequencies of blood or BM cells were analyzed as percentages of total CD45^+^ cells or total BM cells, respectively, as the aim of the analysis was to evaluate the overall hematopoietic alterations induced by the transplantation of *Tet2^-/-^* cells compared to control mice. It is important to note that in control and experimental mice, the contribution of CD45.1 (both from recipient mice as well as competitor cells) and CD45.2 cells in the blood and the BM were different at the time of the analysis, due to the differences in engraftment and expansion of *Tet2*^+/+^ and *Tet2^-/-^* cells.

Histology

Femurs, spleen and liver sections were dissected and kept in 4% PFA at 4°C for 24 hours, after which they were stored in 70% ethanol. The femurs were decalcified in a 10% water solution of EDTA, adjusted to pH 7.4, for two hours and 20 min at 50°C. Then they were stored in fresh 10% EDTA, pH 7.4, for three days at 4°C. Afterwards, they were rinsed under running ionized water for one hour, dehydrated with ethanol, followed by xylene, and subsequently embedded in paraffin. The tissues were sectioned a Leica RM2255 microtome and stained with Hematoxylin/Eosin. Images were acquired with an ECHO Revolve microscope.

Colony formation unit (CFU) assay

Total BM cells (10^4^) were plated in MW6 plates in 2mL of M3434 methylcellulose medium (StemCell Technologies). Cells were incubated at 37 °C for 10 days, after which images of the wells were acquired with a Leica Thunder imager and colonies were counted.

Statistical analysis

Statistical tests and graphical visualization were performed using GraphPad Prism version 9 (GraphPad Prism Software Inc.). Statistical analysis was done with two-tailed Mann-Whitney U test or multiple two-tailed t test for comparison of two groups, and two-way ANOVA with Šidák correction for multiple comparisons when comparing measures over time. The following significance levels were used: ns p>0.05, *p ≤ 0.05, **p ≤ 0.01, ***p ≤ 0.001, ****p ≤ 0.0001. Bar plots show individual values with mean ± standard error of the mean (SEM).

**SUPPLEMENTARY REFERENCES**

1. Quivoron, C. *et al.* TET2 Inactivation Results in Pleiotropic Hematopoietic Abnormalities in Mouse and Is a Recurrent Event during Human Lymphomagenesis. *Cancer Cell* **20**, 25–38 (2011).

2. Ventura, A. *et al.* Restoration of p53 function leads to tumour regression in vivo. *Nature* **445**, 661–665 (2007).

**SUPPLEMENTARY FIGURE LEGENDS**

**Table S1.** Antibodies and dyes for PB analysis

**Table S2.** Antibodies and dyes for BM analysis

**Supplementary figure 1**. Representative flow cytometry gating used for the terminal analysis of immune cell population in PB from transplanted W^41^ mice.

**Supplementary figure 2**. Longitudinal analysis of body weight in mice after BMT. Weight increase of W^41^ mice transplanted with (A) *Tet2*^+/+^ or *Tet2*^−/−^ (CD45.2) total BM cells together with *Tet2*^+/+^ (CD45.1) total BM cells in a 1 to 19 ratio or (B) 1x10^6^ *Tet2*^+/+^ or *Tet2*^−/−^ (CD45.2) total BM cells, without competitor cells. Statistical analysis was done with two-tailed Mann-Whitney U test. The following significance levels were used: ns p>0.05, *p ≤ 0.05, **p ≤ 0.01, ***p ≤ 0.001, ****p ≤ 0.0001. Bar plots show individual values with mean and SEM.

**Supplementary figure 3**. Representative flow cytometry gating used for the terminal analysis of immune cell population in BM from transplanted W^41^ mice.

**Supplementary figure 4**. Terminal flow cytometry analysis of hematopoietic cells in BM from control and CH mice. (A) Quantification of the percentage of CD45.2^+^ cells in PB and BM in transplanted W^41^ mice. (B) Analysis of mature cell populations. Myeloid-derived suppressor cells (MDSC) are represented as the sum of polymorphonuclear MDSC (PMN-MDSC) and mononuclear MDSC (M-MDSC) gated as shown in supplementary figure 3. (C) Analysis of the proportion of CD45.1^+^ and CD45.2^+^ mature immune cells in W^41^ mice from the CH model. Statistical analysis was done with two-tailed Mann-Whitney U test. The following significance levels were used: ns p>0.05, *p ≤ 0.05, **p ≤ 0.01, ***p ≤ 0.001, ****p ≤ 0.0001. Bar plots show individual values with mean and SEM.

**Supplementary figure 5**. Analysis of CFU assay. Representative pictures of a plate well (left) and absolute quantification (right) of the colonies formed after plating 40.000 cells from W^41^ mice transplanted with (A) *Tet2*^+/+^ or *Tet2*^−/−^ (CD45.2) total BM cells together with *Tet2*^+/+^ (CD45.1) total BM cells in a 1 to 19 ratio or (B) 1x10^6^ *Tet2*^+/+^ or *Tet2*^−/−^ (CD45.2) total BM cells, without competitor cells. Statistical analysis was done with two-tailed Mann-Whitney U test. The following significance levels were used: ns p>0.05, *p ≤ 0.05, **p ≤ 0.01, ***p ≤ 0.001, ****p ≤ 0.0001. Bar plots show individual values with mean and SEM.

**Supplementary figure 6**. Experimental design (left) and quantification of *Tet2*^-/-^ CD45.2^+^ cells in PB from W^41^ mice transplanted with 1x10^6^ *Tet2*^-/-^ total BM cells with or without WT CD45.1^+^ competitor cells (right). Bar plots show individual values ± SEM.

**Supplementary figure 7**. Terminal flow cytometry analysis of hematopoietic cells in BM from control and MM mice. (A) Quantification of the percentage of CD45.2^+^ cells in PB and BM in transplanted W^41^ mice. (B) Analysis of mature cell populations. Myeloid-derived suppressor cells (MDSC) are represented as the sum of polymorphonuclear MDSC (PMN-MDSC) and mononuclear MDSC (M-MDSC) gated as shown in supplementary figure 3. (C) Analysis of the proportion of CD45.1^+^ and CD45.2^+^ mature immune cells in W^41^ mice from the MM model. Statistical analysis was done with two-tailed Mann-Whitney U test. The following significance levels were used: ns p>0.05, *p ≤ 0.05, **p ≤ 0.01, ***p ≤ 0.001, ****p ≤ 0.0001. Bar plots show individual values with mean and SEM.
